# Supplementary material for: TRPC6-Mediated Ca2+ Influx Activates MAPK and NFκB Signaling and Elicits Pro-Inflammatory and Catabolic Responses in Human Intervertebral Disc Cells
Source: Cells. 2026 Mar 17;15(6):534. doi: 10.3390/cells15060534 (PMC13025513; doi:10.3390/cells15060534)
Supplement: Supplementary file 1 [file cells-15-00534-s001.zip › cells-4091588-supplementary.pdf]

Supplementary Material

TRPC6-Mediated Ca^{2+} Influx Activates MAPK and NFκB Signaling and Elicits Pro-Inflammatory and Catabolic Responses in Human Intervertebral Disc Cells

Janitri Venkatachala Babu [1], Varun Puvanesarajah [2], Addisu Mesfin [3,4], Jonathan P. Japa [3,4], Kevin Yoon [3,4], Mark Ehioghae [3,4], Michael G. Schrlau [1], Laura S Stone [5], Wolfgang Hitzl [6,7], and Karin Wuertz-Kozak [1,8,*]

1 Department of Biomedical Engineering, Rochester Institute of Technology (RIT), Rochester, NY 14623, USA; jv3384@rit.edu (J.V.B.)

2 Department of Orthopaedics and Physical Performance, University of Rochester Medical Center,

Rochester, NY 14642, USA; varun_puvanesarajah@urmc.rochester.edu

3 MedStar Orthopaedic Institute, MedStar Georgetown University Hospital, Washington, DC 20010, USA;

addisu.mesfin@medstar.net (A.M.); jonathan.p.japa@medstar.net (J.P.J.); kevin.yoon@medstar.net (K.Y.); mark.ehioghae@medstar.net (M.E.)

4 Department of Orthopaedic Surgery, Georgetown University School of Medicine, Washington, DC 20007, USA

5 Department of Mechanical Engineering, Rochester Institute of Technology (RIT), Rochester, NY 14623, USA; mgseme@rit.edu (M.G.S.)

6 Department of Anesthesiology, Faculty of Medicine, University of Minnesota, Minneapolis, MN 55455, USA; stone023@umn.edu

7 Department of Ophthalmology and Optometry, Paracelsus Medical University, 5020 Salzburg, Austria; w.hitzl@salk.at

8 Research Program, Experimental Ophthalmology and Glaucoma Research, Paracelsus Medical University, 5020 Salzburg, Austria

9 Schön Clinic Munich Harlaching, Spine Center, Academic Teaching Hospital and Spine Research Institute of the Paracelsus Medical University 5020 Salzburg (Austria), Munich 81547, Germany

* Correspondence: kwbme@rit.edu* Correspondence: Karin Wuertz-Kozak; kwbme@rit.edu

*Corresponding Author

Karin Wuertz-Kozak

Department of Biomedical Engineering

Rochester Institute of Technology

160 Lomb Memorial Drive, Bldg. 73

Rochester, NY 14623 (USA)

kwbme@rit.edu

Table S1. Human intervertebral disc donor characteristics

Donor Number		Level	Pfirrmann Grade	Diagnosis	Sex	BMI	Age	Steroid injection
1		C4-5	4	Stenosis	Male	25.1	79	No
2		L4-5/ L5-S1	4	DDD	Male	33.6	47	Yes
3		L3-4/ L4-5	4	Stenosis	Female	30.7	75	Yes
4		n.a.	n.a.	n.a.	n.a.	n.a.	n.a.	n.a.
5		n.a.	n.a.	n.a.	n.a.	n.a.	n.a.	n.a.
6		L4-5	5	DDD	Female	30	45	n.a.
7		L5-S1	3	DH	Male	23.8	50	Yes
8		L5-S1	5	DH	Female	38.7	33	Yes
9		L4-5/ L5-S1	4	DH	Male	26	42	Yes
10		C6-7	3	DH	Female	30.8	54	No
11		L5-S1	4	DH	Male	22.3	27	Yes
12		L5-S1	4	DH	Female	32.5	29	Yes
13	●	L5-S1	5	DH	Male	n.a.	36	n.a.
14	■	C5-6	3	DH	Female	n.a.	40	n.a.
15	▲	L5-S1	4	DH	Male	n.a.	41	n.a.
16	◆	L5-S1	3	DH	Female	22.3	36	Yes
17	★	L5-S1	3	DH	Female	20.7	42	Yes

For samples 4 and 5, detailed Pfirrmann grading information was not available; however, based on clinical assessment, these samples fall within the Pfirrmann grade III–V range.

Abbreviations: n.a., not available (clinical or demographic information not provided for these donors); DDD, Degenerative disc disease; DH, Disc herniation.

Table S2. TaqMan gene expression assays used for qPCR analysis

**Nr.**	**Target Gene**	**Gene Name**	**Assay ID**
**1**	*TRPC6*	*Transient receptor potential cation channel subfamily C member 6*	Hs00988479_m1
**2**	*IL6*	*Interleukin 6*	Hs00174131_m1
**3**	*CXCL8 (IL8)*	*C-X-C motif chemokine ligand 8 (interleukin 8)*	Hs00174103_m1
**4**	*PTGS2 (COX2)*	*Prostaglandin-endoperoxide synthase 2*	Hs00153133_m1
**5**	*MMP1*	*Matrix metallopeptidase 1*	Hs00899658_m1
**6**	*MMP2*	*Matrix metallopeptidase 2*	Hs01548727_m1
**7**	*MMP3*	*Matrix metallopeptidase 3*	Hs00968305_m1
**8**	*MMP13*	*Matrix metallopeptidase 13*	Hs00942584_m1
**9**	*ACAN*	*Aggrecan*	Hs00153936_m1
**10**	*ADAMTS4*	*ADAM metallopeptidase with thrombospondin type 1 motif 4*	Hs00192708_m1
**11**	*ADAMTS5*	*ADAM metallopeptidase with thrombospondin type 1 motif 5*	Hs01095518_m1
**12**	*BDNF*	*Brain derived neurotrophic factor*	Hs02718934_s1
**13**	*NGF*	*Nerve growth factor*	Hs00171458_m1
**14**	*VEGFA*	*Vascular endothelial growth factor A*	Hs00900055_m1
**15**	*YWHAZ*	*Tyrosine 3-monooxygenase/tryptophan 5-monooxygenase activation protein zeta*	Hs01122445_g1

All assays were pre-designed TaqMan Gene Expression Assays (FAM-MGB) for Homo sapiens (Thermo Fisher Scientific). Gene expression was normalized to *YWHAZ* and analyzed using the ΔΔCt method.

Table S3. Primary and secondary antibodies used for Western blot pathway analysis

**Nr**	**Antibody**	**Dilution**	**Catalog Nr.**	**Company**
1	α-Tubulin (Rabbit)	1:2000	2144	Cell signaling
2	GAPDH (Rabbit)	1:2000	2118	Cell signaling
3	p44/42 ERK (Mouse)	1:2000	4696	Cell signaling
4	Phospho-p44/42 ERK (rabbit)	1:2000	4370	Cell signaling
5	p38 MAPK (Rabbit)	1:2000	9212	Cell signaling
6	Phospho-p38 MAPK (Mouse)	1:2000	9216	Cell signaling
7	Inhibitor of NF-κB alpha (IKB-α) (Mouse)	1:2000	4814	Cell signaling
8	IRDye® 800CW Goat Anti-Mouse IgG1	1:20,000	926-32350	LICORbio
9	IRDye® 680RD Goat Anti-Rabbit IgG1	1:20,000	926-68071	LICORbio

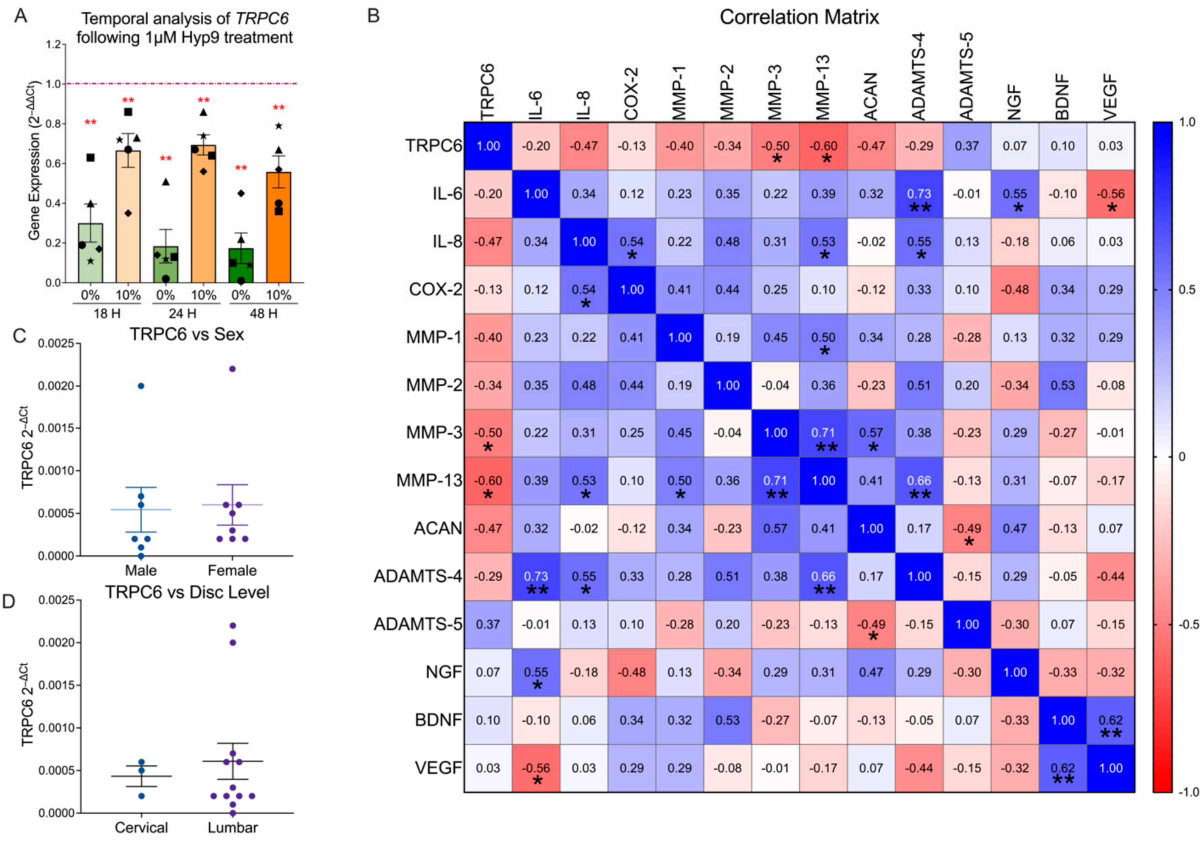

Figure S1. Basal TRPC6 expression and donor-specific associations in human intervertebral disc cells. (A) TRPC6 gene expression under serum-free (0%) and serum-containing (10%) conditions assessed at 18, 24, and 48 h by quantitative PCR following Hyp9 treatment. Data are normalized to YWHAZ and presented as fold change ($2^{-\Delta\Delta Ct}$) relative to vehicle control (dashed line; n = 5 biological replicates). Statistical significance (**$p < 0.01$ vs. vehicle control) was assessed using the Mann–Whitney test. (B) Spearman correlation matrix showing associations between basal TRPC6 expression and selected inflammatory, catabolic, and neuroangiogenic gene transcripts. Correlation coefficients (r) were calculated using TRPC6 $2^{-\Delta Ct}$ values from vehicle control samples (n = 17 donors)

and gene expression values from Hyp9-treated samples (1 μM, 18 h, serum-free; n = 17 donors). Correlation strength is represented by the color scale (−1 to +1), with r values displayed within each cell. Statistical significance is indicated as *p ≤ 0.05, **p ≤ 0.01, and ***p ≤ 0.001 (C) TRPC6 expression stratified by sex. Basal TRPC6 $2^{-\Delta Ct}$ values from vehicle control samples in male and female donors are shown as individual data points with mean ± SEM overlaid. Statistical significance was assessed using the Mann–Whitney test. (D) TRPC6 expression across disc regions. Comparison of basal TRPC6 $2^{-\Delta Ct}$ values from vehicle control samples between cervical and lumbar disc regions. Individual donor values are shown with mean ± SEM overlaid. Statistical significance was assessed using the Mann–Whitney test.

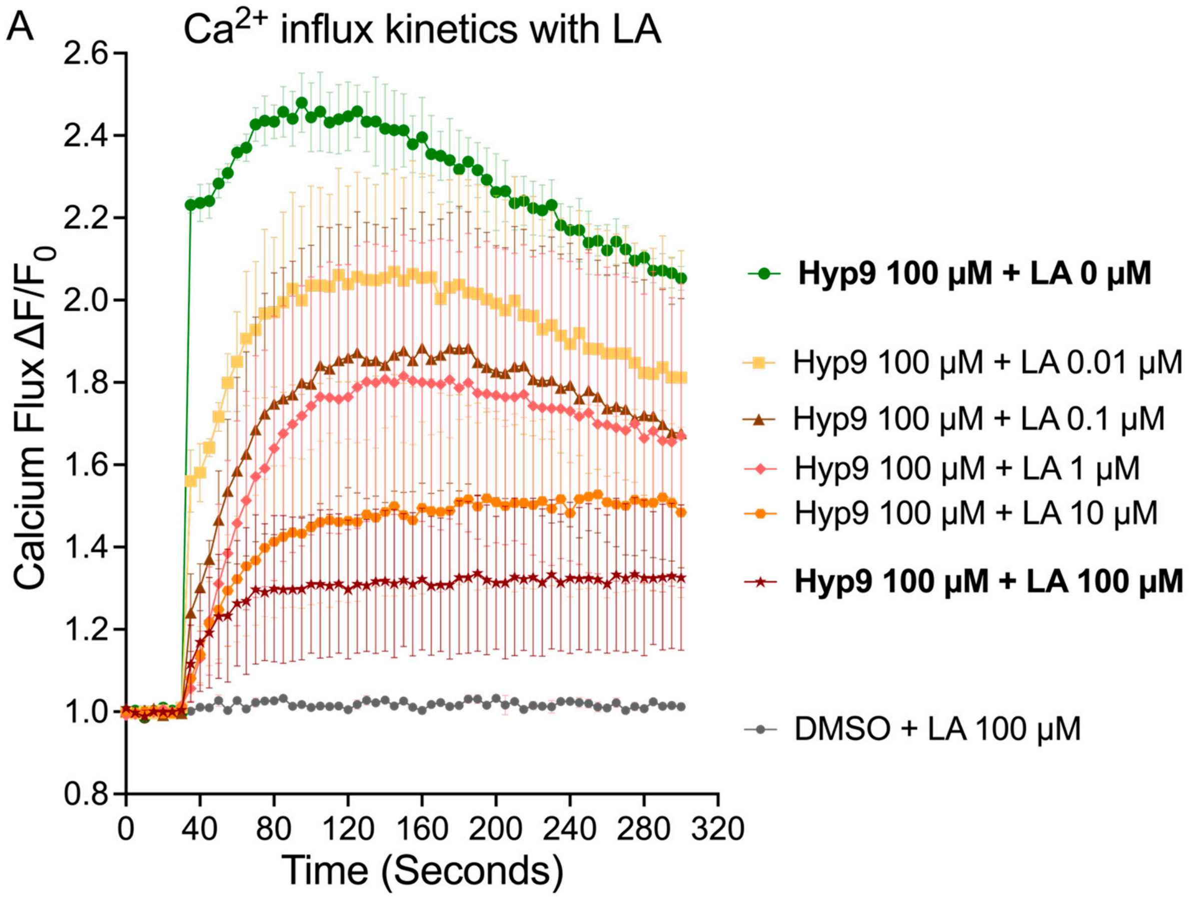

Figure S2. Concentration-dependent attenuation of Hyp9-evoked Ca^{2+} responses by larixyl acetate (LA) in human IVD cells. (A) Representative real-time Ca^{2+} traces ($\Delta F/F_0$) showing inhibition of Hyp9 (100 µM)-induced intracellular Ca^{2+} influx by increasing concentrations of larixyl acetate (LA; 0.01–100 µM). IVD cells were pre-incubated with LA (2X concentration) for 30 minutes prior to dye loading, resulting in the indicated final 1X inhibitor concentrations during measurement. Calcium flux was initiated by automated addition of Hyp9 using the FlexStation 3 platform. Data are presented as mean ± SEM from two independent donors (N = 2). Vehicle (DMSO) controls are shown for comparison.

Table S4. Spearman correlations between basal TRPC6 expression and Hyp9-responsive gene expression.

Nr.	TRPC6 Vs Genes ($2^{-\Delta Ct}$)	Spearman r value	p Value
1	IL-6	-0.29	0.25
2	IL-8	0.08	0.76
3	COX-2	0.10	0.68
4	MMP-1	-0.09	0.73
5	MMP-2	-0.71	0.002
6	MMP-3	0.11	0.66
7	MMP-13	-0.08	0.76
8	ACAN	-0.10	0.72
9	ADAMTS-4	-0.68	0.003
10	ADAMTS-5	-0.15	0.55
11	NGF	-0.19	0.45
12	BDNF	-0.17	0.51
13	VEGF	-0.30	0.22

Spearman correlation coefficients (r) were calculated using TRPC6 expression values expressed as $2^{-\Delta Ct}$ (vehicle control) and gene expression values expressed as $2^{-\Delta\Delta Ct}$ following Hyp9 treatment.

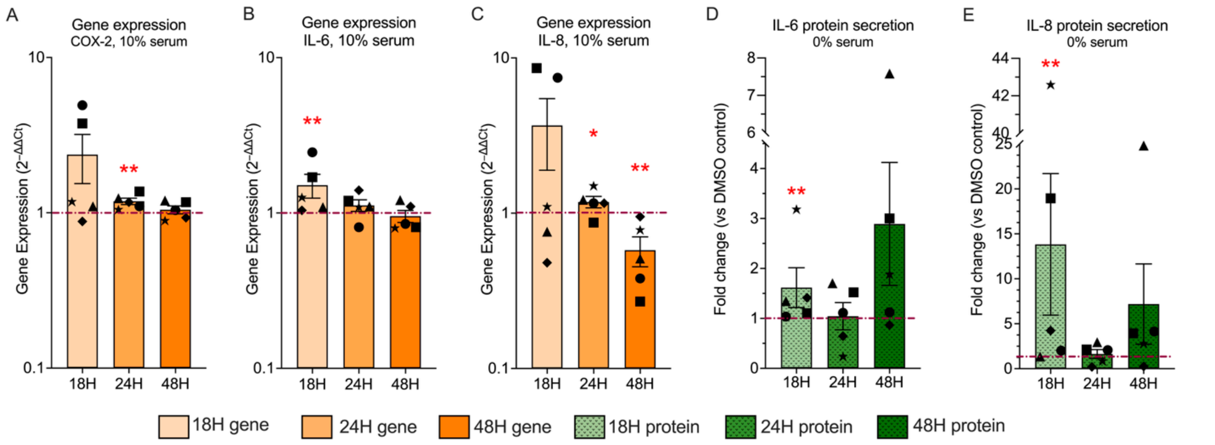

Figure S3. Inflammatory gene expression and protein secretion in human intervertebral disc cells following Hyp9-mediated TRPC6 activation. (A–C) Gene expression of COX-2 (PTGS2) (A), IL-6 (B), and IL-8 (CXCL8) (C) measured at 18, 24, and 48 h under 10% serum conditions by quantitative PCR. mRNA levels are normalized to YWHAZ and presented as fold change ($2^{-\Delta\Delta Ct}$) relative to vehicle control (dashed line; n = 5). (D–E) Protein secretion of IL-6 (D) and IL-8 (E) measured in conditioned media collected at 18, 24, and 48 h under serum-free (0%) conditions. Protein levels were normalized to total DNA content and are presented as fold change relative to vehicle control (dashed line; n = 5). Data are presented as mean ± SEM (biological replicates). Statistical significance (*$p \leq 0.05$, **$p \leq 0.01$, #$p \leq 0.0001$ vs. vehicle control) was assessed using the Mann–Whitney test.

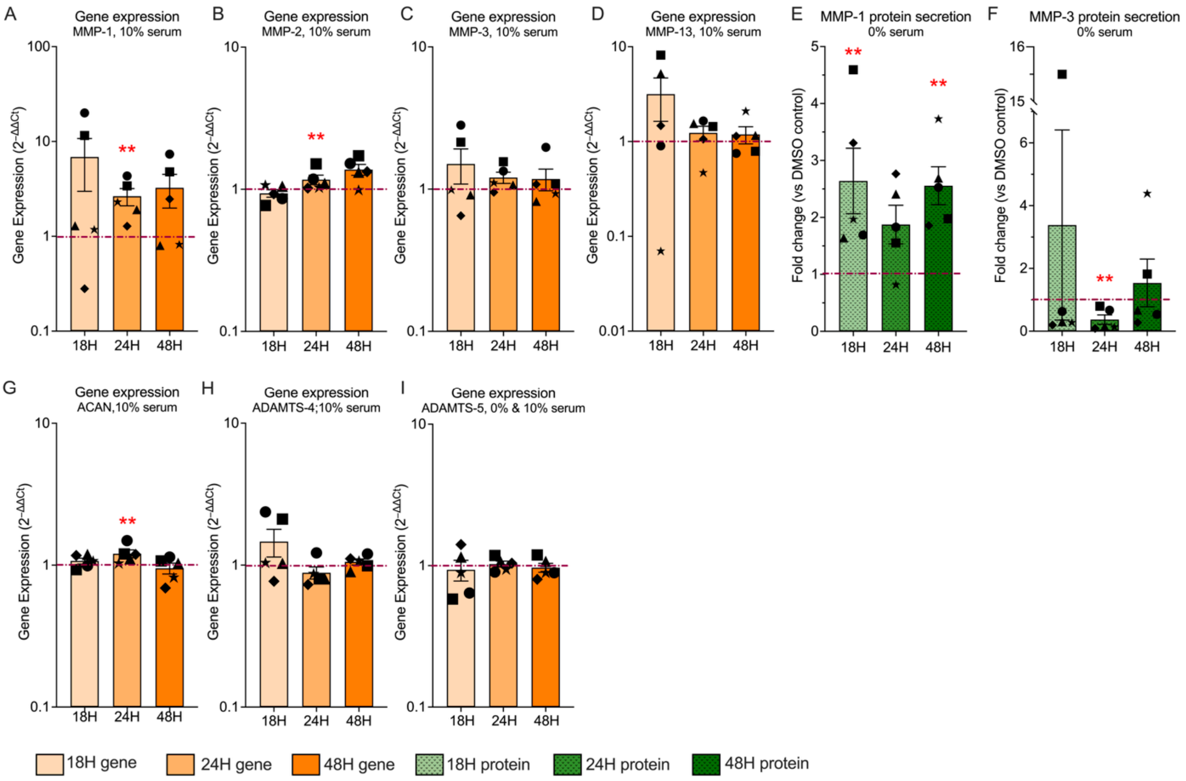

Figure S4. Catabolic gene expression and protein secretion in human intervertebral disc cells following Hyp9-mediated TRPC6 activation. (A–D) Gene expression of MMP-1 (A), MMP-2 (B), MMP-3 (C), and MMP-13 (D) measured at 18, 24, and 48 h under 10% serum conditions by quantitative PCR. mRNA levels are normalized to YWHAZ and presented as fold change ($2^{-\Delta\Delta Ct}$) relative to vehicle control (dashed line; n = 5). (E–F) Protein secretion of MMP-1 (E) and MMP-3 (F) measured in conditioned media collected at 18, 24, and 48 h under serum-free (0%) conditions. Protein levels were normalized to total DNA content and are presented as fold change relative to vehicle control (dashed line; n = 5). (G–J) Gene expression of ACAN (G) ADAMTS-4 (H), and ADAMTS-5 (I) measured at 18, 24, and 48 h under serum-free (0%) and serum-containing (10%) conditions by

quantitative PCR. Data are presented as fold change ($2^{-\Delta\Delta Ct}$) relative to vehicle control (dashed line; n = 5). Data are presented as mean ± SEM (biological replicates). Statistical significance (**p ≤ 0.01, #p ≤ 0.0001 vs. vehicle control) was assessed using the Mann–Whitney test.

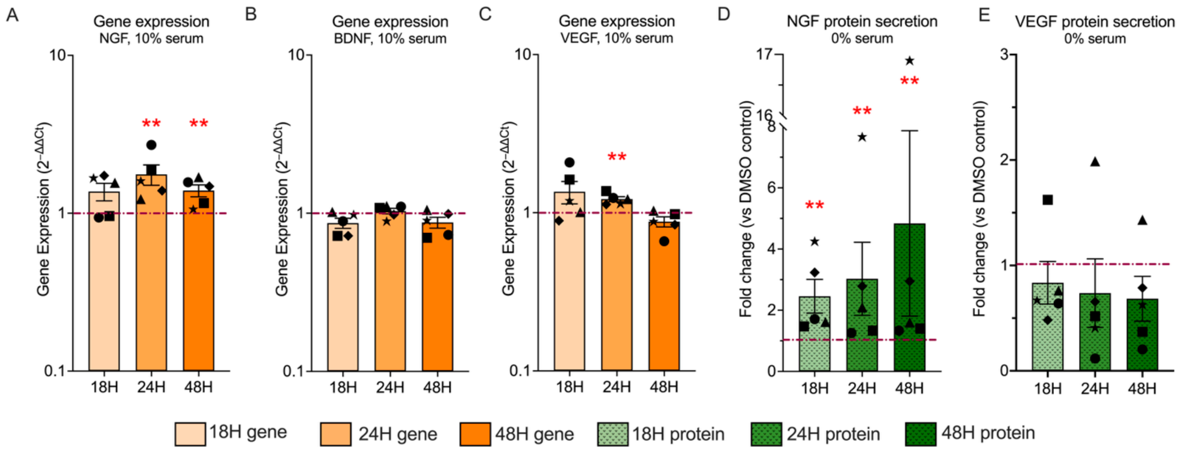

Figure S5. Neuro-angiogenic gene expression and protein secretion in human intervertebral disc cells following Hyp9-mediated TRPC6 activation. (A–C) Gene expression of NGF (A), BDNF (B), and VEGF (C) measured at 18, 24, and 48 h under 10% serum conditions by quantitative PCR. mRNA levels are normalized to YWHAZ and presented as fold change ($2^{-\Delta\Delta Ct}$) relative to vehicle control (dashed line; n = 5). (D–E) Protein secretion of NGF (D) and VEGF (E) measured in conditioned media collected at 18, 24, and 48 h under serum-free (0%) conditions. Protein levels were normalized to total DNA content and are presented as fold change relative to vehicle control (dashed line; n = 5). Data are presented as mean ± SEM (biological replicates). Statistical significance (**p ≤ 0.01 vs. vehicle control) was assessed using the Mann–Whitney test.
